# Ecological Momentary Assessment of Daily Activities in Individuals With Neurological Disorders: Scoping Review

**DOI:** 10.2196/92797

**Published:** 2026-08-14

**Authors:** Eva Delooz, Barbara Piškur, Aki Rintala, Bruno Bonnechère, Annemie Spooren

**Affiliations:** 1 Faculty of Rehabilitation Sciences REVAL Rehabilitation Research Center Hasselt University Diepenbeek, Flanders Belgium; 2 Research Centre for Autonomy and Participation Zuyd University of Applied Sciences Heerlen, Limburg The Netherlands; 3 Physical Activity & Functional Capacity Research Group LAB University of Applied Sciences Lahti, Paijat-Hame Finland; 4 Centre of Expertise in Care Innovation Department of PXL-Healthcare PXL University of Applied Sciences and Arts Hasselt, Flanders Belgium

**Keywords:** ecological momentary assessment, EMA, daily activities, neurological disorders, scoping review

## Abstract

**Background:**

Daily activities shape individuals’ health and well-being, reflecting functioning and lived health. For people with neurological conditions, these activities are often disrupted, affecting autonomy and quality of life. Traditional assessments miss subtle, real-time fluctuations, whereas ecological momentary assessment (EMA) captures moment-to-moment activity within natural contexts, offering insight into person-environment-occupation interactions. Despite its growing use, it remains unclear how EMA protocols conceptualize daily activities and integrate person-environment-occupation dimensions in applications for neurological populations.

**Objective:**

This scoping review aims to map the existing literature on the use of EMA to capture daily activities, ranging from basic self-care to more complex activities, in individuals with neurological disorders.

**Methods:**

A scoping review was conducted to map studies using EMA to capture daily activities in adults with neurological conditions, with a specific focus on content and practical application. A total of 341 articles were identified.

**Results:**

A total of 20 studies using EMA to assess daily activities in neurological populations were included; most were observational, with 2 longitudinal studies and 2 randomized controlled trials. Daily activity questions and response formats varied, often using multiple-choice lists; only 1 study allowed open-ended responses. In addition to daily activity questions, EMA captured person (physical, affective, and cognitive), environment (physical and social), and occupation domains, as well as motivation and EMA disturbance. Protocols differed in setting, schedule, technology, and adherence, with most reporting completion rates above 70%.

**Conclusions:**

EMA captures daily activities in neurological populations and demonstrates generally high adherence despite variability in study designs, questions, and technologies. The findings suggest that the phrasing of EMA items, the predominance of closed-response formats, and the narrow focus on the verb “doing” limit the depth and nuance of the data collected, often overlooking important aspects of performance, engagement, or both in daily activities.

## Introduction

Daily life is made up of countless activities that shape how individuals experience their health and well-being. Understanding how people engage in these daily activities offers important insights into the broader concept of functioning, which has gained recognition as a key link between medical conditions and the lived experience of health [1]. The World Health Organization’s International Classification of Functioning, Disability and Health (ICF) emphasizes functioning not merely as the absence of disease, but as lived health: the capacity to carry out activities and participate in life situations within real-world contexts [2]. This perspective shifts attention toward what individuals are actually able to do in their environments and how they engage with the world around them. However, while the ICF offers a valuable conceptual framework, it provides limited guidance on how personal, environmental, and activity-related factors interact dynamically in daily life. To address this gap, the Person-Environment-Occupation (PEO) model has been widely applied in occupational science and rehabilitation research [3-5]. This model highlights the complex and evolving relationship among the person, environment, and occupation. Meaningful engagement in daily activities arises at the intersection of these 3 domains [4-8]. Daily activities are context- and role-specific, ranging from basic self-care to more complex activities such as work, leisure, and caregiving. They are central to human identity, autonomy, and connectedness [7,9]. Through these activities, individuals express preferences, fulfill social roles, and structure their everyday lives, making engagement in daily activities a vital expression of functioning and lived health. In this review, the term “daily activities” refers to observable actions and tasks performed in everyday life [10]. Although related, this construct is distinct from broader concepts such as participation (involvement in life situations, as defined in the ICF) and occupation (as conceptualized within the PEO model) [2,4]. To ensure conceptual clarity, these terms are used consistently and are not treated as interchangeable.

However, for individuals living with neurological disorders, engagement in daily activities is frequently challenged or disrupted. Neurological disorders encompass a wide range of conditions, including multiple sclerosis (MS), spinal cord injury, stroke, traumatic brain injury, and neurodegenerative disorders such as Parkinson and Alzheimer disease [11]. Recent global data underscore the magnitude of this issue. A major study published in The Lancet Neurology in collaboration with the World Health Organization, drawing on the Global Burden of Disease 2021 dataset, revealed that more than 3 billion people worldwide were living with a neurological disorder in 2021 [12]. Neurological disorders have become the leading cause of disability worldwide. Since 1990, the burden associated with these disorders, measured in disability-adjusted life years—which combine years lost due to ill health, disability, or premature death—has increased by 18%, highlighting their profound impact on functional independence and quality of life [12]. While clinical symptoms such as motor deficits, cognitive decline, and sensory impairments are well documented, the real-world consequences of these disorders, particularly their effect on engagement in daily activities, remain a critical yet underexplored area in both research and clinical practice. For example, motor impairments, such as paralysis and loss of mobility, can disrupt basic self-care activities [13]. A similar pattern is observed in MS, where 42% of individuals reported a decline in their ability to manage daily life over a 2-year period [14]. These conditions differ substantially in symptom presentation, disease progression, and associated functional limitations, influencing the nature of engagement in daily activities.

To effectively support individuals living with neurological disorders, it is essential to gain a nuanced understanding of how daily activities are experienced and performed. To this end, a range of established assessments and observational tools are used to examine the factors that influence engagement in daily activities, namely, the dynamic interaction among the person, the environment, and the activity itself [15]. Within the ICF, a distinction is made between capacity—what a person is able to do in a standardized or controlled environment—and performance—what a person actually does in their real-life context [2]. Assessments at the activity level, such as the Canadian Occupational Performance Measure [16], the Functional Independence Measure, and the Barthel Index [17], primarily address this domain, often with a focus on performance in everyday tasks. Assessments at the participation level of the ICF—referring to a person’s involvement in everyday situations and society—are also limited for individuals with neurological disorders. In addition, diagnosis-specific instruments, such as the Parkinson’s Disease Questionnaire, the Barthel Index, and the Quadriplegia Index of Functioning, are frequently used to capture functional status [2]. However, most of these tools rely on retrospective self-reports and structured observations, which have limited capacity to capture the subtle, moment-to-moment fluctuations that shape everyday functioning [18,19]. As they reflect past experiences rather than real-time activity, they are vulnerable to memory bias, social desirability effects, and inaccurate recall [20,21]. As a result, these methods often fail to detect microprocesses—small but meaningful behavioral and cognitive patterns—that play a crucial role in shaping engagement in daily activities [8,20]. One of the main challenges is the lack of assessments that directly measure how people perform their daily activities. In recent years, advances in digital health technologies have enabled more objective approaches to capturing aspects of daily behavior, such as mobility tracking, sensor-based monitoring, and smartphone interaction data [22]. While these methods provide valuable, continuous measurements of observable behavior, they offer limited insight into the subjective experience, meaning, and contextual factors that shape engagement in daily activities [9,20,22]. As a result, they may not fully capture how individuals perceive, interpret, and engage in their daily lives.

**Figure 1:**
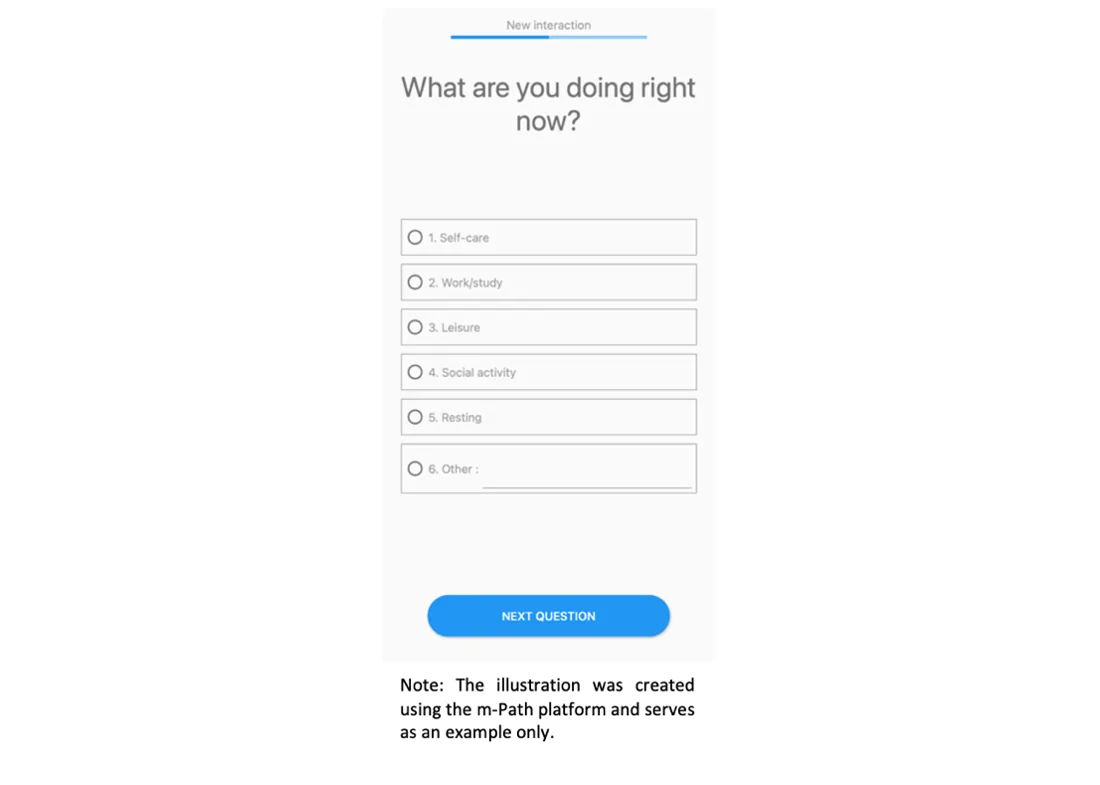
Example of an ecological momentary assessment question.

To overcome the challenge of accurately capturing daily activities, ecological momentary assessment (EMA) has emerged as a promising approach. EMA typically involves repeated assessments administered multiple times per day over several days or weeks, often delivered via smartphone-based apps or other digital devices. Participants receive prompts at fixed or random intervals and are asked to report on their current experiences, behaviors, or context in real time. This approach minimizes recall bias and enhances ecological validity by capturing data in natural environments as events unfold. By collecting real-time data on behavior, emotions, and contextual factors in people’s natural environments through regular, random prompts covering various topics, EMA offers a dynamic way to understand daily life as it unfolds [20,23,24]. Also referred to as ambulatory assessment or experience sampling methodology, EMA has been widely adopted across the health sciences, psychology, and clinical research. Its applications range from monitoring symptoms and evaluating treatments to designing personalized interventions, such as Just-In-Time Adaptive Interventions and Ecological Momentary Interventions, for individuals with and without disabilities [25-30]. To provide a concrete illustration of how EMA is experienced by participants, an example of an EMA prompt is presented in Figure 1. The illustration was created using the m-Path platform and serves as an example only; it was not derived from an included study. The potential of EMA to provide insight into daily activities was recently highlighted in a bibliometric review, which demonstrated that EMA has been applied across a wide range of domains, including neurological disorders. Notably, individuals with neurological disorders represented the third largest population in which daily activities were assessed using EMA [31]. However, while this review provided an overview of the fields in which EMA has been applied, it did not examine how daily activities are specifically measured or how daily activity–related questions, arising from the dynamic interaction among the person, occupation, and environment, are incorporated into EMA protocols for individuals with neurological disorders [31].

In addition to understanding what is measured, it is equally important to consider how EMA protocols that include questions about daily activities are conducted. Key design characteristics of EMA protocols, such as the sampling schedule, number of assessments, delivery tools (eg, smartphone apps), and participant adherence, play a crucial role in determining feasibility, participant burden, and data quality [20,21,24,32]. For example, a higher sampling frequency may increase ecological validity but also participant burden, potentially reducing adherence [32]. Despite the growing use of EMA in health research, there is currently no clear consensus on how these design characteristics should be optimized when assessing daily activities, particularly in populations with neurological disorders. As a result, substantial variability exists in how EMA protocols are applied, which may influence both the feasibility of implementation and the validity of the data collected.

Therefore, an analysis is needed to understand which aspects of daily activities are captured in EMA studies, particularly in relation to the PEO model. For example, it remains unclear whether current EMA methodologies adequately capture person-, environment-, and occupation-related aspects of daily activities. In addition, it is important to understand how EMA is implemented, including its key design characteristics. Therefore, this scoping review aims to map the existing literature on the use of EMA to capture daily activities, ranging from basic self-care to more complex activities, in individuals with neurological disorders. Specifically, this review addresses 2 key dimensions:

- Content-related aspects (WHAT): identifying EMA items that directly assess daily activities and analyzing how additional EMA questions relate to daily activities through the lens of the PEO model; and
- Practical application (HOW): examining the key design characteristics of EMA protocols and their potential implications for feasibility and data quality.

## Methods

### Design

A scoping review was conducted in accordance with the framework of Arksey and O’Malley [33] and reported following the PRISMA-ScR (Preferred Reporting Items for Systematic Reviews and Meta-Analyses Extension for Scoping Reviews; see Multimedia Appendix 1) guidelines to map the existing literature on how EMA is used to capture daily activities in individuals with neurological disorders [34].

### Search Strategy

The search focused on neurological disorders and included the most prevalent conditions identified in Feigin et al [11]. This restriction was applied to ensure methodological coherence and feasibility. To ensure a comprehensive search, we combined the MeSH term “Nervous System Diseases” with a selection of specific neurological conditions. The MeSH term was used in its exploded form, thereby capturing a wide range of related disorders within the hierarchical MeSH structure (eg, cerebrovascular diseases and spinal cord diseases), including conditions not explicitly listed as separate search terms. Additional specific conditions (eg, tetanus, migraine, and brain neoplasms) were included to increase sensitivity, account for potential variability in indexing and terminology across databases, and capture studies not yet indexed with MeSH terms. This set of disorders was combined with terms related to daily activities and EMA. Daily activities were operationalized broadly to capture activities across different contexts and roles, ranging from basic self-care to more complex activities such as work, leisure, productivity, and caregiving. This approach aligns with an occupational perspective in which daily activities are understood as emerging from the dynamic interaction among the person, environment, and occupations performed, as described in the PEO model [3,4]. EMA was defined as a real-time assessment method, also referred to as ambulatory assessment or experience sampling methodology, designed to capture experiences, behaviors, and activities as they occur in everyday life [20,21,23]. The complete search string is presented in Multimedia Appendix 2.

The search was performed on February 27, 2025, across the following electronic databases: MEDLINE, Scopus, Embase, and Web of Science. These databases were selected to ensure broad coverage of biomedical and interdisciplinary research. The search strategies presented in Multimedia Appendix 2 were adapted to the indexing systems and technical requirements of each database. Although minor adjustments in syntax were made, the underlying search concepts and structure remained consistent across databases. The initial search yielded 341 records. After duplicates were removed, 207 unique articles remained. Following the screening of these 207 articles, an additional search for gray literature was performed using DANS Data Station Social Sciences and Humanities, DANS Data Station Life Sciences, and ProQuest Dissertations & Theses. However, this gray literature search did not identify any additional relevant records.

### Data Screening

Following the search strategy, the screening process commenced. An initial screening of titles and abstracts was performed using the following inclusion criteria: (1) studies that used EMA, experience sampling method, or ambulatory assessment, or that met the core characteristics of EMA methodology. These characteristics included repeated data collection in real-world environments using prompts delivered at fixed or random intervals, with participants reporting on their current or very recent experiences, behaviors, or context. Given the variability in terminology, studies were not excluded solely based on the terminology used if their methodological characteristics aligned with EMA. (2) Neurological populations defined according to the classification of neurological disorders described in the Global Burden of Disease framework. Studies were included if the sample consisted predominantly of individuals with a neurological condition. Studies including mixed samples were considered only if data from participants with neurological conditions could be clearly identified. (3) At least one item related to daily activities in the EMA protocol, defined as questions explicitly referring to participants’ engagement in everyday activities or tasks (eg, what the participant was doing at the moment or during a recent period). Only studies including at least one such item within the EMA protocol were retained. (4) Full-text research reports, regardless of publication type (eg, peer-reviewed articles or gray literature such as theses). The exclusion criteria were (1) poster or conference abstracts and (2) mHealth apps that did not implement EMA methodology.

The screening was performed independently by 2 reviewers. Two members of the research group each served as the first reviewer, while the lead researcher (ED) served as the second reviewer. Discrepancies were discussed during regular meetings until consensus was reached. After the initial title and abstract screening, 159 articles were excluded. Subsequently, a full-text screening was conducted, again using independent duplicate review. After this stage, an additional 28 articles were excluded. The final selection comprised 20 articles included for data analysis. The entire screening process was managed using Rayyan software.

### Data Extraction and Analysis

Following screening, the included articles were systematically analyzed based on their full texts. Data analysis was conducted using structured extraction tables developed for this review. Data extraction and coding were performed independently by 2 reviewers, following the same approach used during screening. Any uncertainties or discrepancies regarding extracted variables (eg, PEO categorization, setting, or study type) were discussed during regular meetings until consensus was reached. Three main domains were extracted to address the research question. First, general study characteristics were recorded for each included study, including article information, setting, study design, and population. Second, content-related aspects focused on how daily activities were assessed using EMA. All EMA items directly assessing daily activities were extracted, together with their response formats. In addition, supplementary EMA items were coded according to the components of the PEO model, distinguishing among person-, environment-, and occupation-related constructs. The categorization of EMA variables into the PEO domains was guided by the theoretical definitions of the PEO model [4]. Each item was assigned to the domain that best reflected its primary focus, based on predefined subdimensions (eg, physical, affective, and cognitive aspects for the person domain). Third, practical application aspects addressed key design characteristics of EMA protocols and their potential implications for feasibility and data quality. These data were summarized in a separate extraction table and included the EMA schedule, number and timing of assessments, and reported adherence rates. To explore broader trends in EMA design characteristics, exploratory simple linear regression analyses were conducted using RStudio (version 2024.12.0+467; Posit PBC). Three simple linear regression analyses were performed: (1) regressing the number of assessments per day on study duration (days), (2) regressing adherence on the total number of EMA assessments, and (3) regressing adherence on EMA schedule. Given the limited number of included studies and incomplete reporting of adherence data, the regression analyses were conducted in an exploratory manner to identify potential trends rather than establish confirmatory associations. The analyses were not weighted by sample size. For visualization purposes, study sample size was represented by point size. Model assumptions (eg, linearity, normality of residuals, and homoscedasticity) were assessed by visual inspection. Studies reporting nonfixed ranges for predictor variables were excluded from the relevant regression analyses to avoid arbitrary estimates.

## Results

### General Information of Included Articles

**Figure 2:**
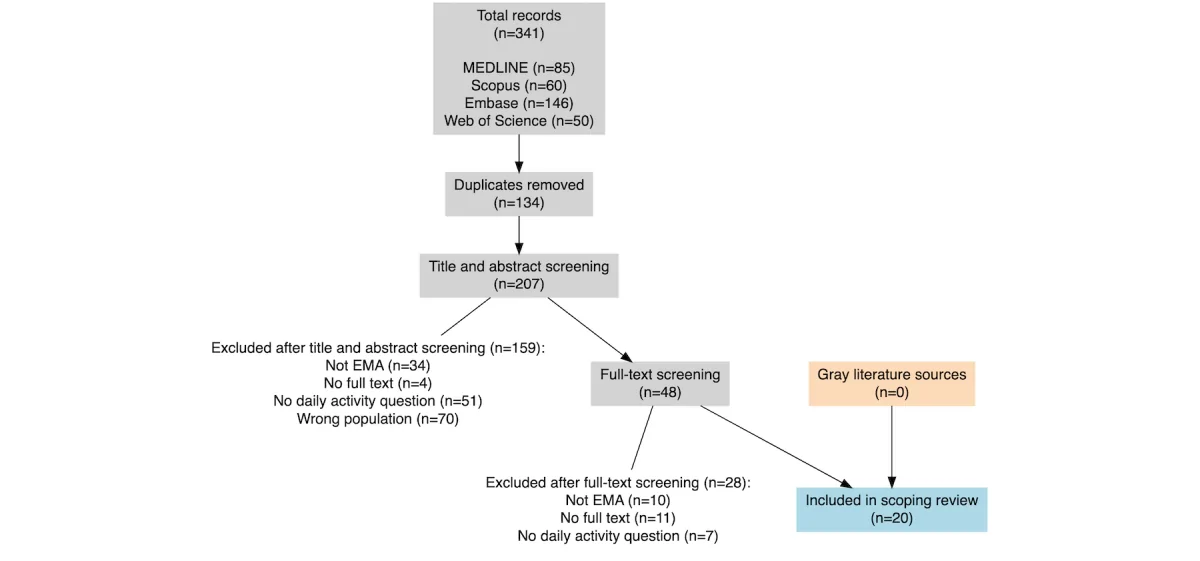
Study flowchart.

In total, 20 articles on the use of EMA to assess daily activities in populations with neurological disorders were included, representing 1128 participants (Figure 2). However, this number should be interpreted with caution, as several publications appeared to originate from overlapping datasets or closely related participant cohorts. Consequently, the reported participant total likely overestimates the number of unique individuals represented across studies. This overlap should also be considered when interpreting patterns in EMA protocol characteristics and adherence. The populations studied included individuals with stroke (n=11), acquired brain injury (n=3), traumatic brain injury (n=2), Parkinson disease (n=1), tinnitus (n=1), hereditary spastic paraplegia (n=1), and MS (n=1). Sample sizes varied considerably across studies, ranging from 7 to 212 participants. The majority of the included studies were observational, alongside 2 longitudinal studies and 2 randomized controlled trials. The studies were published between 2012 and 2024. Most studies (15/20) were conducted primarily in a home setting, often combined with brief laboratory visits. Three studies were conducted entirely in the home environment, 3 exclusively in community settings, 1 exclusively in a hospital, and 1 across both home and hospital settings. An overview of the included studies is presented in Table 1.

**Table 1:** General information about the included studies.

Study	Setting^a^	Study type	Population
Blome et al [35]	Home	Observational (qualitative and exploratory)	People with multiple sclerosis (n=17)
Broen et al [36]	Home	Observational (feasibility study)	Parkinson disease with motor fluctuations (n not stated)
Bui et al [37]	Home + laboratory visits	Observational	Mild-to-moderate stroke (n=202)
Bui et al [38]	Community + laboratory visits	Longitudinal observational	Mild-to-moderate stroke (n=212)
Ezekiel et al [39]	Community (not explicitly stated)	Observational (qualitative usability)	Acquired brain injury (n=7)
Forster et al [40]	Hospital	Observational (pilot study)	Acquired brain injury with cognitive or motor impairments (n=15)
Hart et al [41]	Community	Randomized controlled trial (protocol)	Moderate-to-severe traumatic brain injury (n=60)
Henry et al [42]	Home	Observational (pilot)	Tinnitus (n=24)
Jean et al [43]	Community	Observational (preliminary)	Stroke (first-ever ischemic/hemorrhagic, n=36)
Lau et al [44]	Home + laboratory visits	Observational/prospective cohort	Stroke survivors (n=40, aged 18-65)
Lau et al [45]	Home + laboratory visits	Observational	Stroke survivors (n=40, aged 18-65)
Lau et al [46]	Home + laboratory visits	Observational	Stroke survivors (n=40, aged 18-65)
Lau et al [47]	Home + laboratory visits	Observational	Stroke survivors (n=40, aged 18-65)
Lenaert et al [48]	Home + laboratory visits	Feasibility study/observational	Acquired brain injury (stroke or traumatic brain injury, n=17)
Lenaert et al [49]	Home + laboratory visits	Longitudinal observational	Stroke (n=30)
Sartori et al [50]	Home + laboratory visits	Cross-sectional observational	Hereditary spastic paraplegia (n=50)
Shi et al [51]	Home + laboratory visits	Prospective observational	Stroke (n=202)
Rabinowitz et al [52]	Home + laboratory visits	Randomized controlled trial (exploratory)	Traumatic brain injury (minimum 6 months after injury, n=23)
Villain et al [53]	Hospital (baseline) and home (follow-up)	Prospective observational	Mild ischemic stroke (baseline n=44; follow-up n=34)
Villain et al [54]	Home + laboratory visits	Observational	Minor ischemic stroke (n=34)

^a^Setting refers to the context in which ecological momentary assessments were conducted, as described in the included studies. “Home” indicates assessments primarily conducted in participants’ home environments; “community” refers to assessments conducted in everyday environments beyond the home (eg, during daily life in the community); “hospital” refers to inpatient or clinical settings; and “home + laboratory visits” indicates ecological momentary assessment conducted in daily life combined with scheduled research or clinical visits.

### Content Related

Across the included studies, questions about daily activities differed in both temporal framing and linguistic phrasing (Table 2), yet all were designed to capture participants’ engagement in daily activities. Some studies used explicitly time-bound prompts with multiple-choice response options, such as “What are you doing right now?” [40,42], whereas others referred to a retrospective period, for example, “What have you been doing over the past hour?” [41,52]. The degree of temporal specificity ranged from minutes [39] to hours [41]. By contrast, Lau et al [45] used a general, non-time-specific question (What are you doing?). In terms of phrasing, the verb “doing” was consistently used to denote the performance of or engagement in daily activities. Six studies [39-42,45,52] addressed participants in the second person (you), whereas 3 studies [36,48,49] used first-person phrasing (eg, “What am I doing?”). Notably, in 11 [35,37,38,43,44,46,47,50,51,53,54] of the 20 included articles, the exact wording of the daily activity question was not reported, either in the main manuscript or in the available supplementary materials.

**Table 2:** Content-related daily activities questions and answer possibilities.

Study	Questions on daily activities	Answer possibilities	
		Multiple choice answers	Open box
Blome et al [35]	Usual activities (question not reported)	A lot of daily activity options (options not reported)	Not reported
Broen et al [36]	Currently I am:	Resting, working, housework, hygiene, eating, drinking, relaxing, conversating, or other	Not reported
Bui et al [37]	Daily activities (question not reported)	Physical, cognitive, social, activities of daily living, instrumental activities of daily living, vocational, or passive leisure activities. Further grouped into “total productive” versus “nonproductive, sedentary” activities.	Not reported
Bui et al [38]	Daily activities (question not reported)	Physical, cognitive, social, activities of daily living, instrumental activities of daily living, vocational, or passive leisure activities. Further grouped into “total productive” versus “nonproductive, sedentary” activities.	Not reported
Ezekiel et al [39]	What have you been doing for the last 10 minutes?	Looking after myself, sport exercise, working or studying, social activities, using a computer/tablet/phone, household or family tasks, hobby or quiet pastime, or traveling	Not reported
Forster et al [40]	What are you doing right now?	Exercising, therapy, drinking coffee, taking a break, watching TV, eating, listening to radio/music, reading, surfing the internet, talking to others, or being outside	Not reported
Hart et al [41]	What have you been doing over the past hour or so?	Learning/school activity, working, household chores/pet care, leisure activity, pray/worship/meditate, resting/sleeping, watching TV/video, walking, shopping/errands, talking or socializing, reading, listening to music/radio/podcast, playing videogames/e-games, active play/sports/exercise, intimate relations, social media/internet/email/texting, preparing food/cooking, care of children, or other	Not reported
Henry et al [42]	What are you doing right now?	Working at paid job, watching TV/computer, etc, eating a meal, physical activity, or other	Not reported
Jean et al [43]	Current activity (question not reported)	Doing nothing or resting, work, sports, watching television, listening to music, having an in-person conversation, having a phone conversation, nonphysical leisure, personal hygiene, eating, or other daily life activities	Not reported
Lau et al [44]	Current daily activity (question not reported)	Multiple choice options were not reported.	Not reported
Lau et al [45]	What are you doing?	List of 44 daily activities, developed based on validated ecological momentary assessment surveys and the Activity Card Sort. According to the Activity Card Sort, activities were categorized into 4 domains (instrumental activities of daily living [eg, shopping], high-physical-demand leisure activities [eg, gardening], low-physical-demand leisure activities [eg, watching TV], and social activities [eg, visiting family or friends]), or an additional domain of activities of daily living (eg, toileting).	Not reported
Lau et al [46]	Daily activities (question not reported)	Multiple choice options were not reported.	Not reported
Lau et al [47]	Current activity (question not reported)	List of 44 daily activities (eg, watching TV, eating out, gardening), developed based on validated ecological momentary assessment surveys and the Activity Card Sort.	Not reported
Lenaert et al [48]	What am I doing (just before the beep)? And also? And?	Doing nothing, resting, work, household, self-care, taking care of partner, relaxation, or something else	Not reported
Lenaert et al [49]	What am I doing?	Nothing, resting, working, household, self-care, relaxing, traveling, or other	Not reported
Sartori et al [50]	Ongoing activity (question not reported)	Not reported	Categorized into productive, leisure, interactions, personal care, and others (open-ended)
Shi et al [51]	What activities they were engaging in (question not reported)	Productive (eg, eating, exercising, visiting health care providers) or nonproductive (eg, resting, smoking, and doing nothing). Options were not reported.	Not reported
Rabinowitz et al [52]	What have you been doing over the past hour or so?	Learning/school activity working, household chores/pet care, leisure activity, pray/worship/meditate, resting/sleeping, watching TV/video, walking, shopping/errands, talking or socializing, reading, listening to music/radio/podcast, playing videogames/e-games, active play/sports/exercise, intimate relations, social media/internet/email/texting, preparing food/cooking, care of children, or other (to be specified in textbox)	Not reported
Villain et al [53]	Activities of daily living (question not reported)	Leisure activities (sport, nonphysical leisure, watching television, or listening to music) and activities of daily living (working, shopping, cooking, or housework). Options were not reported.	Not reported
Villain et al [54]	Activities (question not reported)	Multiple choice options were not reported.	Not reported

As presented in Table 2, various response formats were used to assess daily activities. As many as 19 [35-49,51-54] of the 20 included studies relied on multiple-choice response options with predefined activities, whereas 1 study [50] allowed open-ended responses. Several studies offered highly detailed lists of daily activities, often based on validated instruments. For instance, Lau et al [45,47] presented a list of 44 discrete activities derived from validated EMA surveys and the Activity Card Sort, which were subsequently grouped into 4 functional domains—instrumental activities of daily living, high-physical-demand leisure, low-physical-demand leisure, and social activities—with an additional category for basic activities of daily living [45,47]. Bui et al [37,38] also used a multidimensional classification, grouping activities into physical, cognitive, social, activities of daily living, instrumental activities of daily living, vocational, and passive leisure activities, and further categorizing them as “total productive” versus “nonproductive/sedentary” [37,38]. Forster et al [40], Henry et al [42], Ezekiel et al [39], Rabinowitz et al [52], and Hart et al [41] provided varied lists encompassing both work-related and leisure activities, including digital media use (eg, listening to music, social media, and gaming) and household responsibilities (eg, cooking and caring for children). Additionally, 3 studies [43,48,49] explicitly included the option “doing nothing.” The option “other” was included in 6 studies; however, it was not always clear whether participants could provide a written specification for this response or whether it simply indicated that none of the listed options applied [36,41-43,49,52]. In 5 studies, the specific multiple-choice response options were not reported in either the main manuscript or the supplementary material [44,46,50,53,54]. The complete set of detailed data is presented in Table 2.

Alongside items related to daily activities, the EMA protocols included measures of various additional constructs, as daily activities were not the primary focus of all included studies. To support synthesis and interpretation, these constructs were classified using the PEO model. Within this review, daily activities are conceptually understood as emerging from the dynamic interaction among the person, environment, and occupation, consistent with occupational science perspectives. However, for the purpose of this supplementary analysis, the PEO model was applied analytically by examining its components separately. This approach was used to categorize EMA items that addressed person-, environment-, or occupation-related aspects without constituting direct assessments of daily activities. Although the PEO domains are inherently interrelated, considering them as distinct analytical categories enabled the identification of common patterns and areas of emphasis across studies (Table 3).

**Table 3:** Additional questions related to components of the PEO^a^ model.

Study	Person				Environment			Occupation
	Physical	Cognitive	Affective	Physical		Social		
Blome et al [35]	Mobility and pain discomfort	Not reported	Anxiety and depression	Not reported		Not reported	Self-care	
Broen et al [36]	Parkinson-related symptoms and somatic	Not reported	Mood	Contextual factors (location)		Contextual factors (social presence and comfort)	Event related: importance of the event, pleasure	
Bui et al [37]	Not reported	Not reported	Depressed mood	Current location		Social company	Activity quality and satisfaction of the activity	
Bui et al [38]	Poststroke symptoms	Not reported	Not reported	Current location		Social company	Not reported	
Ezekiel et al [39]	Reaction time (objective indicator of fatigue) and energy levels	Not reported	Not reported	Not reported		Not reported	Not reported	
Forster et al [40]	Not reported	Self-reflection frequency	Mood	Not reported		Social context	Judgment of performance	
Hart et al [41]	Not reported	Not reported	PANAS^b^ items (eg, scared, alert, nervous)	Location		Social context	Enjoyment of the activity and sense of accomplishment	
Henry et al [42]	Tinnitus perception	Not reported	Happiness-sadness and anxiety-calmness	Location and loudness of the context		Social context	Not reported	
Jean et al [43]	Not reported	Not reported	Not reported	Immediate location		Social company	Not reported	
Lau et al [44]	Not reported	Not reported	Basic psychological needs (autonomy, competence, and relatedness)	Not reported		Not reported	Not reported	
Lau et al [45]	Physical fatigue and pain	Cognitive complaints and mental fatigue	Depressed mood and cheerfulness	Not reported		Not reported	Not reported	
Lau et al [46]	Symptoms of stroke	Cognitive complaints	Depressed and cheerful affect	Not reported		Not reported	Not reported	
Lau et al [47]	Physical fatigue and pain	Cognitive complaints and mental fatigue	Depressed mood	Not reported		Not reported	Not reported	
Lenaert et al [48]	Pain, fatigue, and not feeling well	Not reported	Mood and self-esteem	Location		Social context	Important event that happened, pleasant activity, control over activity, unexpected activity, and important activity	
Lenaert et al [49]	Physical well-being, current fatigue, and physical activity	Not reported	Perceived effort, enjoyment, and mood	Location		Not reported	Enjoyment of the activity and perceived effort	
Sartori et al [50]	Not reported	Cognitive	Affective	Location, environmental challenges, and skills		Social context	Environmental challenges and skills	
Shi et al [51]	Pain, tiredness, and stress	Concentration	Anxiety, worthlessness, and cheerfulness	Location		Social isolation and company	Not reported	
Rabinowitz et al [52]	Not reported	Not reported	PANAS items (eg, scared, alert, nervous)	Physical context		Social context	Enjoyment of the activity and accomplishment of activity	
Villain et al [53]	Not reported	Not reported	Mood and depression symptoms	Not reported		Not reported	Not reported	
Villain et al [54]	Not reported	Not reported	Anxiety and depression symptoms	Not reported		Not reported	Not reported	

^a^PEO: Person-Environment-Occupation.

^b^PANAS: Positive and Negative Affect Schedule.

Within the person domain, the PEO model distinguishes among physical, affective, and cognitive dimensions. Physical aspects were frequently assessed across the included studies and included symptoms such as pain, fatigue, mobility limitations, stress, and somatic complaints [35,36,38,42,44-49,51]. One study [39] also incorporated objective performance indicators, such as reaction time, as a proxy for fatigue. Affective measures were particularly prevalent, capturing mood states ranging from positive (eg, cheerfulness) to negative (eg, anxiety, depression, and feelings of worthlessness). Emotional valence was assessed using either discrete affective descriptors (eg, Positive and Negative Affect Schedule [PANAS] items) or global mood ratings. Cognitive constructs included concentration, cognitive complaints, mental fatigue, self-reflection frequency, and perceived effort (eg, Lau et al [45-47], Forster et al [40]; Sartori et al [50]; Shi et al [51]), although these were assessed far less frequently than physical or affective dimensions [40,45-47,50,51].

Within the environment domain, additional EMA items were categorized into physical, institutional, cultural, and social environments according to the PEO model. Only the physical and social environments were represented in the included studies; no EMA items addressed institutional or cultural environments. The physical environment was typically assessed through questions about participants’ immediate location [37,38,41-43,48-51] and environmental conditions such as noise or loudness [36,42]. Assessments of the social environment focused on the presence or absence of social company, perceived social support, and experiences of social isolation [36-38,40-43,48,50-52].

Within the occupation domain, considerably fewer EMA items addressed this aspect than the person and environment domains. In the PEO framework, occupation is typically divided into self-care, productivity, and leisure. Only 1 study [35] explicitly assessed self-care activities. The remaining studies addressed the occupation domain more broadly by focusing on constructs such as activity quality, perceived importance of the event, satisfaction with the activity, judgment of performance, sense of accomplishment, perceived control over the activity, and whether the activity was considered important or unexpected [36,37,40,41,48-50,52].

In addition to the constructs categorized according to the PEO model, some EMA measures could not be directly assigned to a single PEO component, including motivation and EMA disturbance. In 4 studies [44,46,47,50], motivation was assessed alongside PEO-related constructs, consistent with self-determination theory. One study [48] also assessed EMA disturbance, defined as the extent to which the EMA protocol itself was perceived as disruptive during daily life. The complete set of detailed data on these additional constructs is presented in Table 3.

### Practical Application

Across the included studies, a variety of technological platforms were used, including the PsyMate app (n=3), the RealLife Exp app (n=2), the PIEL Survey App (n=4), EQ-5D-AA on a mobile phone, custom Android apps, movisensXS, Palm Pilot, and PMAT v2.1.2. EMA schedules also varied considerably across studies. The complete set of detailed data is presented in Table 4.

**Table 4:** Practical application.

Study	EMA^a^ schedule	EMA tool	Time points	Adherence, %
Blome et al [35]	3 times/day for 9 days (morning, midday, and evening)	EQ-5D-AA on mobile phone	27	92.6
Broen et al [36]	10 times/day for 5 days, semirandom within 90-minute blocks (7:30 AM-10:30 PM)	PsyMate app (iPod Touch or own smartphone)	50	84.0
Bui et al [37]	5 times/day for 14 days between fixed time blocks (8 AM-10 PM)	Not specified	70	86.3
Bui et al [38]	5 times/day for 14 days, random start within fixed 2-hour blocks	Status/Post app (iPod Touch or iPhone)	70	84.0
Ezekiel et al [39]	8 times/day for 6 days; 2 fixed + 6 stratified random (10 AM-8 PM)	Custom Android app	48	Not reported
Forster et al [40]	8 times/day for 7 days, semirandom (8 AM-8 PM)	movisensXS (Android)	56	71.6
Hart et al [41]	5 times/day, pseudo-randomized over a 14-hour window. Duration in days not reported.	RealLife Exp app	Not reported	Not reported
Henry et al [42]	4 times/day for 14 days, fixed intervals with reminders	Palm Pilot (CERTAS software)	56	90
Jean et al [43]	5 times/day for 7 days, fixed time blocks per participant	PMAT version 2.1.2 (PDA)	35	74.1
Lau et al [44]	8 times/day, 7 days, 2-hour intervals (8 AM-10 PM)	PIEL Survey App	56	93.6
Lau et al [45]	8 times/day, 7 days, 2-hour intervals (8 AM-10 PM)	PIEL Survey App	56	93.6
Lau et al [46]	8 times/day, 7 days, 2-hour intervals (8 AM-10 PM)	PIEL Survey App	56	93.6
Lau et al [47]	8 times/day, 7 days, 2-hour intervals (8 AM-10 PM)	PIEL Survey App	56	93.6
Lenaert et al [48]	10 signals/day for 6 days, semirandom (7:30 AM-10:30 PM)	PsyMate	60	71.2
Lenaert et al [49]	10 signals/day for 6 days, semirandom (7:30 AM-10:30 PM)	PsyMate	60	65
Sartori et al [50]	6-8 times/day for 7 days, randomized (8 AM-10 PM)	Not specified	42-56	54.9
Shi et al [51]	5 times/day for 14 days, about 2.5-hour intervals (8 AM-10 PM)	iPod Touch or iPhone app	70	84.3
Rabinowitz et al [52]	5 times/day, pseudo-random within a 14-hour window, 7-18 days	RealLife Exp app	35-90	65
Villain et al [53]	5 times/day, 7 days (fixed blocks: 9 AM-10 PM)	Palm Tungsten E2	35	74
Villain et al [54]	5 times/day for 7 days	Palm Tungsten E2	35	74

^a^EMA: ecological momentary assessment.

**Figure 3:**
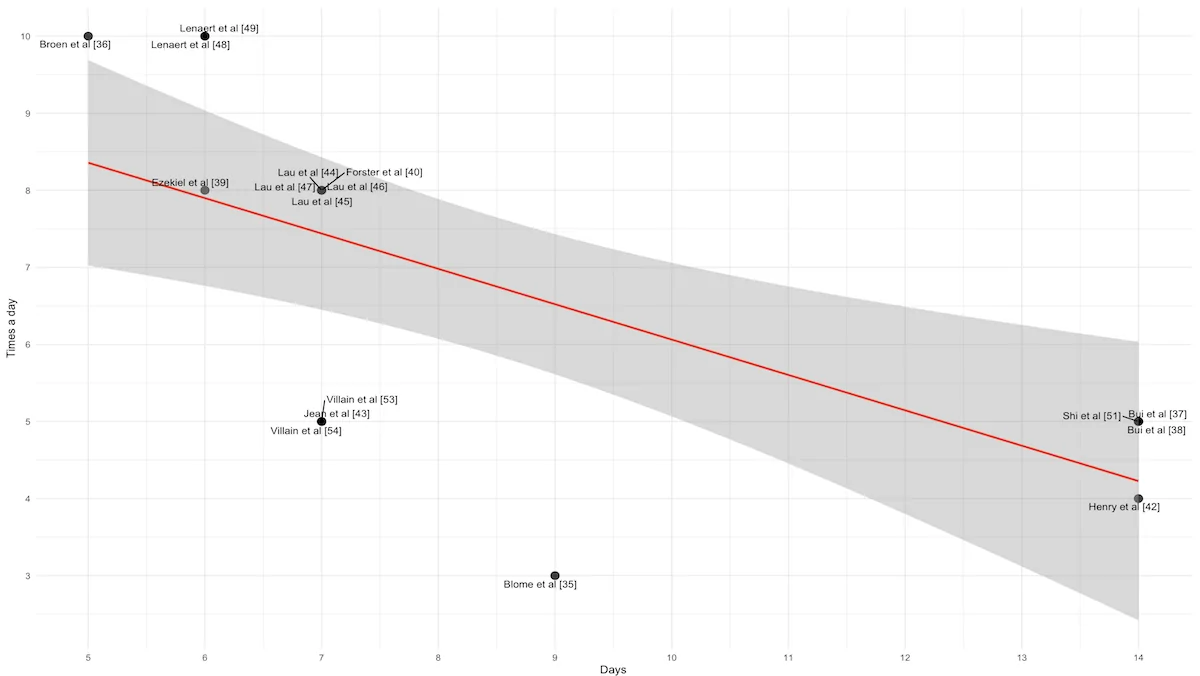
Regression analysis of the ecological momentary assessment schema of the included studies.

Given the variability in EMA schedules, Figure 3 (also see [35-40,42-49,51,53,54]) provides a visual representation of the included protocols. Regarding prompt timing within the day, 3 main scheduling patterns were identified: fixed intervals (eg, Henry et al [42]), semirandom or pseudorandom prompts within predefined time blocks (eg, Broen et al [36] and Lenaert et al [48,49]), and mixed fixed and random approaches (eg, Ezekiel et al [39]). The number of daily prompts also varied substantially across studies, ranging from fixed schedules (eg, Lau et al [46]) to variable schedules reported as ranges (eg, 6-8 prompts/day in Sartori et al [50]; 7-14 prompts/day in Rabinowitz et al [52]). Studies reporting a range rather than a fixed number of daily prompts were excluded from the exploratory regression analyses to avoid arbitrary estimates. In addition, Hart et al [41] was excluded because of insufficient reporting on EMA duration. Consequently, the exploratory regression analyses included 17 studies. The number of daily prompts ranged from 3 to 10, whereas study duration ranged from 5 to 14 days. Some studies combined shorter study periods with high sampling intensity. For instance, Broen et al [36] and Lenaert et al [48,49] collected data over only 5 days but prompted participants up to 10 times per day, representing the highest daily sampling intensity. Conversely, Henry et al [42], Shi et al [51], and Bui et al [37,38] collected data over 14 days but limited daily prompts to 4 or 5. A significant inverse association was observed (β=–.46, *P*=.004): studies with shorter durations generally used higher daily sampling frequencies, whereas those with longer study periods used fewer daily assessments. A slightly different pattern was observed in Blome et al [35], which used a 9-day protocol with 3 daily prompts. Approximately half of the included studies used a 7-day protocol with 5-8 prompts per day [40,43-47,50,53,54].

**Figure 4:**
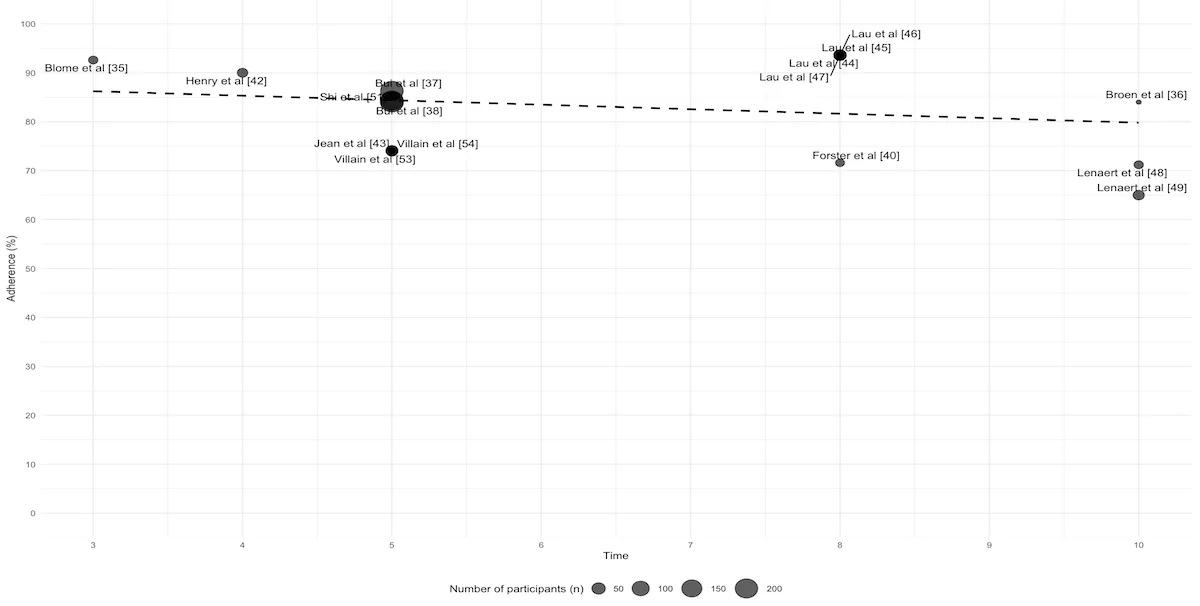
Adherence rates in relation to the total prompts a day.

**Figure 5:**
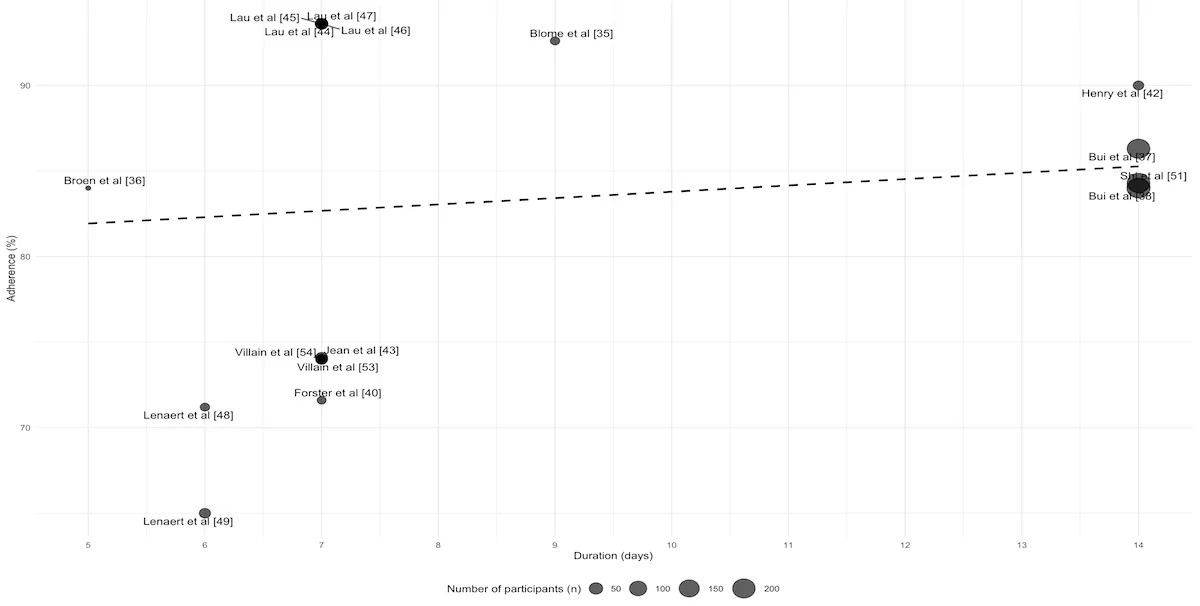
Adherence rates in relation to the total number of days.

**Figure 6:**
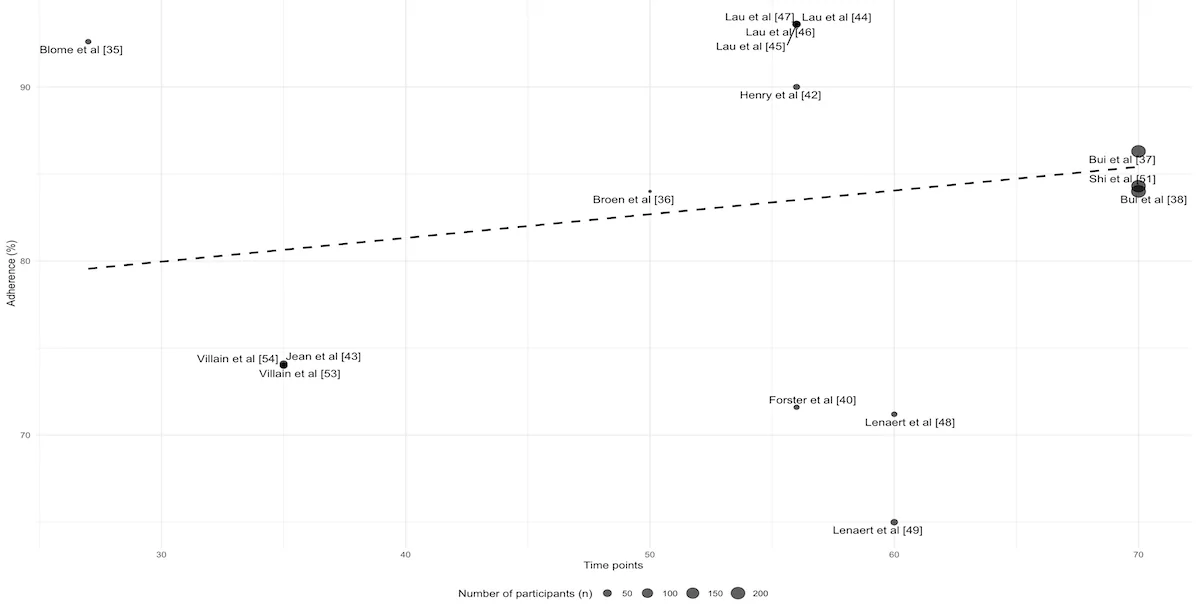
Adherence rates in relation to the total number of time points.

Additionally, adherence rates were examined in relation to the number of daily prompts. As shown in Figure 4 (see also [35-38,40,42-49,51,53,54]), adherence rates varied widely across studies, ranging from approximately 55% to more than 90%. No clear association was observed (β=–.919, *P*=.42). A slight positive trend was observed in Figure 5 ([see also 35,36,40-49,51-54]) between adherence and study duration, with longer studies tending to show somewhat higher adherence. However, this relationship did not reach statistical significance (β=.851, *P*=.28). Adherence rates were also examined in relation to the total number of EMA assessments (Figure 6; see also [35-38,40,42-49,51,53,54]). Overall, adherence was relatively high, with most studies reporting completion rates above 70%. The highest adherence (93.6%) was reported in studies with 56 assessments (eg, Lau et al [45-47]), whereas 1 study [35] reported similarly high adherence (92.6%) with 27 assessments. No significant linear association was observed between the total number of assessments and adherence (β=.087, *P*=.66). However, studies with a high number of assessments (>65) generally maintained adherence above 80% (eg, Bui et al [37] and Shi et al [51]), whereas studies with 50-60 assessments showed greater variability, with completion rates ranging from 55% to more than 90%. For consistency across the exploratory regression analyses, only studies reporting the complete set of required variables were included. Four studies were excluded because of incomplete reporting of adherence, EMA duration, or the total number of assessments. Specifically, Hart et al [41] and Ezekiel et al [39] did not report adherence data, whereas Rabinowitz et al [52] and Sartori et al [50] reported variable EMA schedules without a fixed study duration. To ensure comparability across analyses, these studies were excluded from the regression models and are therefore not represented in Figures 4-6. These findings should be interpreted as exploratory because of the limited number of included studies.

## Discussion

### Principal Findings

This scoping review aimed to map the existing literature on the use of EMA to capture daily activities in individuals with neurological disorders, with a specific focus on content-related aspects and practical application. Twenty articles published between 2012 and 2024 were identified in which EMA was used to assess daily activities. Although EMA has been available for several decades since its introduction by Csikszentmihalyi and Larson in 1987 [21], only 20 studies [35-54] have focused on this population with the explicit aim of gaining insight into daily activities [55,56]. This relatively small body of literature is surprising because improving daily activities is a central goal of neurological rehabilitation and is essential for maximizing recovery, maintaining independence, and supporting long-term health, participation, and quality of life after formal rehabilitation has ended [2,9,57]. Given that EMA is specifically designed to capture experiences and behaviors in everyday life, its application to assessing daily activities in neurological rehabilitation remains surprisingly limited.

While EMA holds clear promise as a method for capturing daily activities, it is important to distinguish it from emerging passive sensing approaches. Although technologies such as wearable sensors and smartphone-based tracking can objectively capture aspects of behavior (eg, movement, location, or device use), they do not provide direct insight into the meaning, intention, or subjective experience of daily activities [22]. EMA uniquely captures these experiential and contextual dimensions, which are central to understanding engagement in daily activities from a PEO perspective.

The aim of this review was not only to consider the theoretical potential of EMA but also to examine how it is currently being applied to gather information about everyday life in individuals with neurological disorders, with the goal of identifying opportunities for broader and more effective use. EMA protocols appropriately vary according to the research aims and the daily-life phenomena being captured. Nevertheless, transparent reporting of the specific EMA items used is crucial. This review revealed that such transparency was often lacking, hindering interpretation and comparison across studies. As a result of the limited number of EMA studies focusing specifically on daily activities, we also included studies in which this construct was assessed as a secondary outcome to map its current operationalization. This may explain why daily activity items were often not described in detail, as they were not the primary focus of many included studies. In addition, a key principle of EMA is its ability to capture experiences and behaviors in the moment, thereby minimizing retrospective bias. Yet, several studies framed their questions retrospectively, for example, by asking participants, “What have you been doing in the past hour?” [52]. This approach undermines one of the central advantages of EMA, highlighting a gap between its theoretical potential and its practical implementation [20,21,24]. These findings also point to the need for more structured guidance on the design and reporting of EMA protocols. In particular, greater consistency in the timing, phrasing, and content of EMA items would improve comparability across studies and enhance interpretability. Developing a core set of reporting recommendations or a checklist for EMA studies assessing daily activities in neurological populations may therefore be a valuable next step.

This limitation in temporal framing also intersects with how daily activities are conceptualized in EMA studies. Questions frequently rely on the verb “doing” to capture engagement in daily activities. Although Wilcock [58] described doing as a core dimension of human occupation—alongside being, becoming, and, later, belonging—this perspective suggests that focusing primarily on “doing” may not fully capture the complexity of engagement in daily activities among individuals with neurological disorders. When physical performance is restricted, such a focus may overlook other meaningful forms of engagement, such as planning, initiating, or contributing to daily activities. From a theoretical perspective, doing represents only 1 dimension of the broader concept of engagement. A narrow emphasis on doing may therefore overlook aspects such as occupational deprivation and occupational alienation. Although these concepts were not directly assessed in the included studies, the observed patterns in EMA item content can be interpreted in light of established occupational science frameworks. For example, occupational deprivation refers to a state in which individuals are prevented from participating in meaningful occupations because of external constraints, such as environmental barriers, social restrictions, or health-related limitations [10,59]. Similarly, occupational alienation occurs when engagement in daily activities lacks meaning, purpose, or personal satisfaction, even when the activity is physically possible [10]. By focusing primarily on “doing,” EMA protocols may overlook the depth and richness of information that can be obtained. However, incorporating other PEO elements—personal factors, environmental contexts, and characteristics of the occupation itself—can help offset the limitations of focusing solely on “doing” and provide more meaningful insights into daily activities [4,10,60].

The way an EMA question is phrased directly shapes the type of responses it elicits. The multiple-choice response options used in the included studies resemble a classification system—such as self-care, leisure, or productivity—rather than providing insight into how a daily activity is performed or how the individual is engaged in that activity. While such categories may serve a useful descriptive purpose, they often remain at the level of occupational type rather than capturing the richer dimensions of daily activity. This limited framing can also obscure the so-called dark side of occupation—activities that may be harmful, socially undesirable, or associated with negative consequences [61]. However, some studies included an “other” response option, which could, in theory, capture such activities. Similarly, activities such as “doing nothing” are rarely explored in depth, even though they may represent diverse states such as rest, disengagement, or lack of opportunity. Recent developments in EMA methodology suggest that open-ended response formats can address some of these limitations. Open-ended questions are increasingly being used in other fields to capture richer data—for example, in autism research, where participants may respond not only through writing but also through drawing, providing insight into nuances that predefined categories may not capture [62,63]. Although open-ended EMA questions require more time and effort to complete, they can generate a broader range of responses and uncover insights that researchers may not have anticipated [63,64]. In this context, the value of open-ended questions lies in their ability to reveal the complexity, individuality, and sometimes unexpected aspects of daily activities that might otherwise remain hidden.

In addition to items targeting daily activities, many EMA protocols incorporated a range of supplementary questions. Classification of these questions using the PEO model revealed that a substantial proportion pertained to person- and environment-related factors. The strong representation of person-related factors is consistent with the findings of a recent bibliometric analysis examining the use of EMA to capture daily activities across health and disability populations [31]. With respect to environmental factors, a pronounced imbalance was observed. Most questions addressed the physical and social environment, whereas the cultural and institutional dimensions were largely absent. Institutional factors, such as organizational routines, policies, or the physical environment of institutions, received little attention despite evidence from neurological rehabilitation research demonstrating their relevance to participation and engagement [65]. Although the limited assessment of institutional factors may partly reflect the predominance of home-based EMA studies, in which these factors are less salient, this gap is more concerning in ambulatory and inpatient settings. Similarly, the cultural environment remains underrepresented in EMA research on daily activities among individuals with neurological disorders. For example, sociological research has highlighted the importance of incorporating cultural dimensions to achieve a more comprehensive understanding of human behavior and experience [66]. Expanding EMA methodologies to systematically address both cultural and institutional contexts could enhance the ecological validity of findings and provide a more comprehensive representation of the environments in which daily activities occur. However, one could argue that these contexts are relatively stable throughout the day and therefore may not require frequent assessment using EMA. At the same time, other environmental dimensions, such as the virtual environment, are becoming increasingly relevant in contemporary life, yet none of the included studies assessed this aspect [3].

Beyond the content-related aspects, this review also provides insight into the practical application of EMA for capturing daily activities. The results indicate consistently high adherence rates, supporting the feasibility of EMA in this context. Similar patterns have been reported in other populations. For example, a scoping review in perinatal mental health reported an average compliance rate of approximately 80%, ranging from 63% to 96%, indicating strong feasibility [67]. In populations with psychosis, a systematic review found a mean survey completion rate of 67%, with more than 86% of participants meeting minimum engagement criteria [68]. Likewise, a cross-diagnostic study reported median adherence rates above 80%, with early response behavior strongly predicting sustained participation over time [69]. Collectively, these findings suggest that well-designed EMA protocols—carefully balancing sampling frequency, participant burden, and interface usability—can achieve high adherence, supporting both comprehensive data collection and sustained participant engagement. However, the available data do not allow conclusions regarding the optimal number of assessments per day or the ideal duration of EMA protocols. Beyond adherence, this review also identified substantial variation in EMA design characteristics, including the number of daily prompts, study duration, sampling schedules, and response windows. Although these findings provide an overview of how EMA is currently implemented to assess daily activities in neurological populations and may inform the design of future studies, the available evidence remains insufficient to determine which protocol configurations are most effective for optimizing feasibility and data quality.

### Limitations

The findings of this review should be interpreted in light of several methodological limitations. First, the search strategy may have been subject to selection bias. Although a broad search strategy was applied, including the use of the MeSH term “Nervous System Diseases,” variability in indexing and the hierarchical structure of MeSH may have influenced the retrieval of specific conditions. As a result, some conditions were captured implicitly, whereas others were included explicitly, reflecting a balance between sensitivity and specificity. Searches were conducted using EMA-related terminology in the title and abstract, which may have resulted in the omission of relevant studies that used similar methodologies without explicitly using these terms. Although studies were screened for core EMA characteristics whenever possible, some eligible publications may not have been identified. Consequently, the size and scope of the available evidence base may have been underestimated. In addition, although this approach aligns with the exploratory nature of a scoping review, the absence of an extended manual search strategy (eg, reference checking or citation tracking), beyond the gray literature search, may have further limited the comprehensiveness of the evidence base. Incorporating such strategies in future reviews could yield additional insights. Second, this scoping review was conducted in accordance with the framework proposed by Arksey and O’Malley [33] and reported following the PRISMA-ScR guidelines, strengthening its methodological rigor and transparency. Nevertheless, the limitations related to the search strategy and the variability in reporting across the included studies should be considered when interpreting the findings. Third, the predefined inclusion criteria may have introduced conceptual ambiguity. The identification of EMA studies was based on the presence of terms such as ecological momentary assessment, experience sampling methodology, or ambulatory assessment in the title or abstract. However, the operationalization of EMA varies substantially across studies, particularly with respect to sampling frequency, duration, and intensity (eg, once daily vs multiple prompts per day). As a result, studies using markedly different methodological approaches were grouped under a single EMA label. Future research would benefit from a more explicit definition of EMA to improve conceptual clarity and comparability across studies. Furthermore, although 20 studies were included, several publications originated from the same research groups. This suggests that similar theoretical and methodological perspectives may have informed multiple articles. It also cannot be ruled out that overlapping datasets were used, as many studies relied on comparable samples and study parameters. Although definitive conclusions cannot be drawn, the body of evidence may therefore represent fewer than 20 fully independent perspectives. This observation highlights the importance of considering not only the number of studies but also the diversity of methodological approaches when interpreting the findings. Finally, substantial heterogeneity was observed across the included studies with respect to sample size, study duration, the number of EMA prompts, and adherence reporting. This variability complicates direct comparisons across studies and limits the extent to which the findings can be synthesized beyond a descriptive level, consistent with the aims of a scoping review. Furthermore, the regression analyses should be interpreted as exploratory because of the heterogeneity among studies, the limited number of included studies, and the incomplete reporting of adherence data.

### Future Directions

Therefore, future studies should prioritize greater transparency in reporting EMA protocols and work toward developing more standardized guidelines or checklists to support consistency in EMA design and reporting. Researchers are encouraged to move beyond a narrow focus on “doing” and explore alternative question formats—including open-ended or mixed-format items—that can capture richer, more nuanced information about the context, meaning, and personal relevance of daily activities. In addition, when using predefined activity categories, it is important to include response options that allow participants to report less commonly addressed or potentially negative occupations, acknowledging that not all daily activities are inherently positive. Future research should also investigate the conditions under which adherence is highest to better understand the factors that support sustained engagement in EMA studies.

Methodologically, participatory approaches—such as co-designing EMA items with individuals who have lived experience of neurological disorders—may help ensure that questions reflect the diversity and complexity of real-world engagement. Combining EMA with complementary qualitative methods (eg, brief interviews or activity diaries) could further deepen insights and capture aspects of occupational engagement that might otherwise be overlooked. These strategies would enable EMA to more fully reflect the dynamic interplay among personal factors, environmental contexts, and occupations, ultimately broadening its value in both research and clinical practice.

### Conclusions

This review systematically mapped both what EMA captures about daily activities in individuals with neurological disorders and how EMA is implemented in this context. Regarding the content of EMA protocols, the findings showed that daily activities were predominantly assessed using items focused on the verb “doing” and mainly closed-response formats. Additional EMA questions addressed several components of the PEO model, although physical and social environmental dimensions were represented more frequently than cultural and institutional dimensions. Together, these findings suggest that current EMA approaches may provide a relatively narrow representation of daily activity performance and engagement, potentially overlooking important nuances of individuals’ lived experiences. Regarding practical application, considerable variability was observed in EMA protocol characteristics, including assessment frequency, study duration, prompting schedules, and adherence rates. Although adherence was generally high across studies, the heterogeneity of protocols and the exploratory nature of the available evidence make it difficult to draw firm conclusions about which design characteristics best support feasibility and data quality in neurological populations. Rather than identifying an optimal EMA approach, this review provides an overview of how EMA is currently being applied and highlights areas requiring further investigation. Future EMA research in neurological populations would benefit from greater transparency in reporting EMA items, broader use of open-ended response formats, and more systematic consideration of underrepresented environmental dimensions. In addition, studies directly comparing EMA protocol characteristics are needed to better understand how protocol design influences feasibility, adherence, ecological validity, and the quality of data collected for individualized assessment of daily activities.

## Data Availability

This scoping review does not include original data or materials, as it synthesizes and analyzes findings from previously published studies. Therefore, no datasets or study materials are available for sharing.

## Appendix

Multimedia Appendix 1 The PRISMA-ScR (Preferred Reporting Items for Systematic Reviews and Meta-Analyses Extension for Scoping Reviews) checklist.

Multimedia Appendix 2 Search string.

## Abbreviations

EMA: ecological momentary assessment
ICF: International Classification of Functioning, Disability and Health
MS: multiple sclerosis
PANAS: Positive and Negative Affect Schedule
PEO: Person-Environment-Occupation
PRISMA-ScR: Preferred Reporting Items for Systematic Reviews and Meta-Analyses Extension for Scoping Reviews

## References

[1] Boggs D, Polack S, Kuper H, Foster A (2021). Shifting the focus to functioning: essential for achieving Sustainable Development Goal 3, inclusive Universal Health Coverage and supporting COVID-19 survivors. Glob Health Action.

[2] World Health Organization (WHO) (2001). International Classification of Functioning, Disability and Health. WHO.

[3] Hinojosa P, Kramer C, Royeen CB (2017). Perspectives on Human Occupations: Theories Underlying Practice.

[4] Law M, Cooper B, Strong S, Stewart D, Rigby P, Letts L (2016). The Person-Environment-Occupation Model: a transactive approach to occupational performance. Can J Occup Ther.

[5] Strong S, Rigby P, Stewart D, Law M, Letts L, Cooper B (1999). Application of the Person-Environment-Occupation Model: a practical tool. Can J Occup Ther.

[6] Hagedorn R (2000). Tools for Practice in Occupational Therapy: A Structured Approach to Core Skills and Processes.

[7] Hammell KRW (2014). Belonging, occupation, and human well-being: an exploration. Can J Occup Ther.

[8] Unsworth C (2007). Research in occupational therapy: methods of inquiry for enhancing practice. Aus Occup Therapy J.

[9] Wilcock A (2006). An Occupational Perspective of Health.

[10] Townsend E, Polatajko H (2007). Enabling Occupation II: Advancing an Occupational Therapy Vision for Health, Well-being, & Justice Through Occupation.

[11] Feigin VL, Vos T, Nichols E, Owolabi MO, Carroll WM, Dichgans M, Deuschl G, Parmar P, Brainin M, Murray C (2020). The global burden of neurological disorders: translating evidence into policy. The Lancet Neurology.

[12] GBD 2021 Nervous System Disorders Collaborators (2024). Global, regional, and national burden of disorders affecting the nervous system, 1990-2021: a systematic analysis for the Global Burden of Disease Study 2021. Lancet Neurol.

[13] Yetzer Elizabeth, Blake Karen, Goetsch Nancy, Shook Mary, St Paul Marilyn (2017). SAFE medication management for patients with physical impairments of stroke, part two. Rehabil Nurs.

[14] Bass Ann D, Van Wijmeersch Bart, Mayer Lori, Mäurer Mathias, Boster Aaron, Mandel Matt, Mitchell Colin, Sharrock Kersten, Singer Barry (2020). Effect of multiple sclerosis on daily activities, emotional well-being, and relationships: The Global vsMS Survey. Int J MS Care.

[15] Law M, Baptiste S, Carswell A, McColl MA, Polatajko H, Pollock N (2014). Canadian Occupational Performance Measure 5th ed. (COPM).

[16] Law M, Baptiste S, McColl M, Opzoomer A, Polatajko H, Pollock N (1990). The Canadian occupational performance measure: an outcome measure for occupational therapy. Can J Occup Ther.

[17] Cardol M, de Haan RJ, van den Bos GA, de Jong BA, de Groot IJ (1999). The development of a handicap assessment questionnaire: the Impact on Participation and Autonomy (IPA). Clin Rehabil.

[18] AOTA (2020). Occupational Therapy Practice Framework: domain and process—fourth edition. American Journal of Occupational Therapy.

[19] Nielsen KT, Wæhrens Eva Ejlersen (2015). Occupational therapy evaluation: use of self-report and/or observation?. Scand J Occup Ther.

[20] Shiffman S, Stone AA, Hufford MR (2008). Ecological momentary assessment. Annu Rev Clin Psychol.

[21] Csikszentmihalyi M, Larson R (1987). Validity and reliability of the Experience-Sampling Method. J Nerv Ment Dis.

[22] Trifan A, Oliveira M, Oliveira JL (2019). Passive sensing of health outcomes through smartphones: systematic review of current solutions and possible limitations. JMIR Mhealth Uhealth.

[23] Moskowitz DS, Young SN (2006). Ecological momentary assessment: what it is and why it is a method of the future in clinical psychopharmacology. J Psychiatry Neurosci.

[24] Stone A, Shiffman S (1994). Ecological momentary assessment (EMA) in behavioral medicine. Annals of Behavioral Medicine.

[25] Hall M, Scherner PV, Kreidel Y, Rubel JA (2021). A systematic review of momentary assessment designs for mood and anxiety symptoms. Front Psychol.

[26] Kwasnicka D, Kale D, Schneider V, Keller J, Yeboah-Asiamah Asare B, Powell D, Naughton F, Ten Hoor Gill A, Verboon P, Perski O (2021). Systematic review of ecological momentary assessment (EMA) studies of five public health-related behaviours: review protocol. BMJ Open.

[27] Mink F, Hehlmann M, Lutz W (2024). Ecological momentary assessment in clinical research: a systematic review. ResearchGate.

[28] Perski O, Keller J, Kale D, Asare BY, Schneider V, Powell D, Naughton F, Ten Hoor Gill, Verboon P, Kwasnicka D (2022). Understanding health behaviours in context: a systematic review and meta-analysis of ecological momentary assessment studies of five key health behaviours. Health Psychol Rev.

[29] Schneider S, Junghaenel DU, Smyth JM, Fred Wen CK, Stone AA (2024). Just-in-time adaptive ecological momentary assessment (JITA-EMA). Behav Res Methods.

[30] Mei X, Li Y, Yeung WF, Hu Y, Li J, Li M, Yorke J (2025). Effectiveness of ecological momentary interventions on pain, mental health, and quality of life in individuals with rheumatic diseases: a systematic review and meta-analysis of randomized controlled trials. J Nurs Manag.

[31] Delooz E, Bonnechère Bruno, Piškur Barbara, Spooren A (2026). Tracking daily activities with ecological momentary assessment: a bibliometric analysis of current use in health Mapping daily activities with ecological momentary assessment. PLOS Digit Health.

[32] Stinson L, Liu Y, Dallery J (2022). Ecological momentary assessment: a systematic review of validity research. Perspect Behav Sci.

[33] Arksey H, O'Malley L (2005). Scoping studies: towards a methodological framework. International Journal of Social Research Methodology.

[34] Moher D, Liberati Alessandro, Tetzlaff Jennifer, Altman Douglas G, PRISMA Group (2009). Preferred reporting items for systematic reviews and meta-analyses: the PRISMA statement. Ann Intern Med.

[35] Blome C, Carlton J, Heesen C, Janssen MF, Lloyd A, Otten M, Brazier J (2021). How to measure fluctuating impairments in people with MS: development of an ambulatory assessment version of the EQ-5D-5L in an exploratory study. Qual Life Res.

[36] Broen MPG, Marsman VAM, Kuijf ML, Van Oostenbrugge RJ, van Os J, Leentjens AFG (2016). Unraveling the relationship between motor symptoms, affective states and contextual factors in Parkinson's disease: a feasibility study of the experience sampling method. PLoS One.

[37] Bui Q, Kaufman KJ, Munsell EG, Lenze EJ, Lee J, Mohr DC, Fong MW, Metts CL, Tomazin SE, Pham V, Wong AW (2024). Smartphone assessment uncovers real-time relationships between depressed mood and daily functional behaviors after stroke. J Telemed Telecare.

[38] Bui Q, Kaufman KJ, Pham V, Lenze EJ, Lee J, Mohr DC, Fong MW, Metts CL, Tomazin SE, Wong AW (2022). Ecological momentary assessment of real-world functional behaviors in individuals with stroke: a longitudinal observational study. Arch Phys Med Rehabil.

[39] Ezekiel L, Veiga JJD, Ward T, Dawes H, Collett J (2023). Exploring the usability of a smartphone application to monitor fatigue and activity for people with acquired brain injury. Br J Occup Ther.

[40] Forster SD, Gauggel S, Petershofer A, Völzke Volker, Mainz V (2020). Ecological momentary assessment in patients with an acquired brain injury: a pilot study on compliance and fluctuations. Front Neurol.

[41] Hart T, Rabinowitz A, Vaccaro M, Chervoneva I, Wilson J (2019). Behavioral activation augmented with mobile technology for depression and anxiety in chronic moderate-severe traumatic brain injury: protocol for a randomized controlled trial. Arch Rehabil Res Clin Transl.

[42] Henry JA, Galvez G, Turbin MB, Thielman EJ, McMillan GP, Istvan JA (2012). Pilot study to evaluate ecological momentary assessment of tinnitus. Ear Hear.

[43] Jean FAM, Swendsen JD, Sibon I, Fehér Kristoffer, Husky M (2013). Daily life behaviors and depression risk following stroke: a preliminary study using ecological momentary assessment. J Geriatr Psychiatry Neurol.

[44] Lau SC, Connor LT, Baum CM (2023). Associations between basic psychological need satisfaction and motivation underpinning daily activity participation among community-dwelling survivors of stroke: an ecological momentary assessment study. Arch Phys Med Rehabil.

[45] Lau SC, Connor LT, King AA, Baum CM (2022). Multimodal ambulatory monitoring of daily activity and health-related symptoms in community-dwelling survivors of stroke: feasibility, acceptability, and validity. Arch Phys Med Rehabil.

[46] Lau SCL, Connor LT, Skidmore ER (2024). Associations of circadian rest-activity rhythms with affect and cognition in community-dwelling stroke survivors: an ambulatory assessment study. Neurorehabil Neural Repair.

[47] Lau SC, Connor LT, Skidmore ER, King AA, Lee J, Baum CM (2023). The moderating role of motivation in the real-time associations of fatigue, cognitive complaints, and pain with depressed mood among stroke survivors: an ecological momentary assessment study. Arch Phys Med Rehabil.

[48] Lenaert B, Colombi M, van Heugten C, Rasquin S, Kasanova Z, Ponds R (2019). Exploring the feasibility and usability of the experience sampling method to examine the daily lives of patients with acquired brain injury. Neuropsychol Rehabil.

[49] Lenaert B, Neijmeijer M, van Kampen N, van Heugten C, Ponds R (2020). Poststroke fatigue and daily activity patterns during outpatient rehabilitation: an experience sampling method study. Arch Phys Med Rehabil.

[50] Sartori RD, Marelli M, D'Angelo MG, Delle Fave A (2019). Autonomy level and quality of everyday experience of people with hereditary spastic paraplegia. Health Soc Care Community.

[51] Shi Y, Fong MW, Metts CL, LaVela SL, Bombardier C, Hu L, Wong AW (2024). Dynamics of perceived social isolation, secondary conditions, and daily activity patterns among individuals with stroke: a network analysis of ecological momentary assessment data. Arch Phys Med Rehabil.

[52] Rabinowitz A, Hart T, Wilson J (2021). Ecological momentary assessment of affect in context after traumatic brain injury. Rehabil Psychol.

[53] Villain M, Sibon I, Renou P, Poli M, Swendsen J (2017). Very early social support following mild stroke is associated with emotional and behavioral outcomes three months later. Clin Rehabil.

[54] Villain M, Sibon I, Renou P, Poli M, Swendsen J (2021). Depression and routinization following stroke. Rev Neurol (Paris).

[55] Larson R, Csikszentmihalyi M (2014). Flow and the foundations of positive psychology. The Experience Sampling Method.

[56] Prescott S, Csikszentmihalyi M (1981). Environmental effects on cognitive and affective states: the experiential time sampling approach. Soc Behav Pers.

[57] Barker RN, Sealey CJ, Polley ML, Mervin MC, Comans T (2017). Impact of a person-centred community rehabilitation service on outcomes for individuals with a neurological condition. Disabil Rehabil.

[58] Ross MW (2005). Typing, doing, and being: sexuality and the internet. J Sex Res.

[59] Whiteford G (2000). Occupational deprivation: global challenge in the new millennium. British Journal of Occupational Therapy.

[60] Christiansen C, Townsend E (2013). Introduction to Occupation: The Art of Science and Living.

[61] Twinley R (2013). The dark side of occupation: a concept for consideration. Aust Occup Ther J.

[62] Chen Y, Xi Z, Greene T, Mandy W (2025). A systematic review of ecological momentary assessment in autism research. Autism.

[63] Stadel M, Langener Am, Hoemann K, Bringmann Lf (2025). Assessing daily life activities with experience sampling methodology (ESM): scoring predefined categories or qualitative analysis of open-ended responses?. Methods in Psychology.

[64] van Roekel Eeske, Keijsers Loes, Chung Joanne M (2019). A review of current ambulatory assessment studies in adolescent samples and practical recommendations. J Res Adolesc.

[65] Beck M, Engelke E, Birkelund R, Martinsen B (2020). Becoming a nomad when hospitalized with a neurological disease: a phenomenological study. International Journal of Qualitative Studies on Health and Well-being.

[66] Lohani M, Blodgett G (2025). Innovative and ecological: integrating ecological momentary assessment into environmental science research. Front Psychol.

[67] Zhang X, Cui W, Yao J, Zhang Y, Wang Y (2025). Feasibility and utility of ecological momentary assessment to measure mental health issues in perinatal women: scoping review. Psychiatry Res.

[68] Bell Imogen H, Eisner Emily, Allan Stephanie, Cartner Sharla, Torous John, Bucci Sandra, Thomas Neil (2024). Methodological characteristics and feasibility of ecological momentary assessment studies in psychosis: a systematic review and meta-analysis. Schizophr Bull.

[69] Jones SE, Moore RC, Pinkham AE, Depp CA, Granholm E, Harvey PD (2021). A cross-diagnostic study of adherence to ecological momentary assessment: comparisons across study length and daily survey frequency find that early adherence is a potent predictor of study-long adherence. Pers Med Psychiatry.

