# Supplementary material for: Ecological Momentary Assessment of Daily Activities in Individuals With Neurological Disorders: Scoping Review
Source: JMIR Rehabil Assist Technol. 2026 Aug 14;13:e92797. doi: 10.2196/92797 (PMC13525364; doi:10.2196/92797)
Supplement: Supplementary file 2 [file rehab_v13i1e92797_app2.docx]

**Search string**

("Daily functioning"[Tiab] or "Daily activitie*"[Tiab] OR "Daily activity*"[Tiab] OR "Activities of Daily Living"[Mesh] OR "Activities of Daily Living"[Tiab] OR "Basic Activities of Daily living"[Tiab] OR "Leisure"[Tiab] OR "self-care"[Tiab] OR "productivity"[Tiab] OR "Performance of daily activities" [Tiab] OR "occupational performance"[Tiab] OR "meaningful activitie*"[Tiab] OR "participation"[tiab] OR "Social Participation"[Mesh] OR "Social Participation"[tiab]) AND ("Ecological Momentary Assessment"[Mesh] OR "ecological momentary assessment"[Tiab] OR "Experience sampling method"[Tiab] OR "Ambulatory assessment"[Tiab] OR "Experience sampling"[Tiab] OR "Real-time assessment"[Tiab] OR "Mobile Based EMA"[Tiab]) AND ("Nervous System Diseases"[Mesh] OR "Nervous System Diseases"[tiab] OR "Tetanus"[Mesh] OR "Tetanus"[tiab] OR "Meningitis"[Mesh] OR "Meningitis"[tiab] OR "Encephalitis"[Mesh] OR "Encephalitis"[tiab] OR "Epilepsy, Complex Partial"[Mesh] OR "Epilepsy, Complex Partial"[tiab] OR "Motor Neuron Disease"[Mesh] OR "Motor Neuron Disease"[tiab] OR "Migraine Disorders"[Mesh] OR "Migraine Disorders"[tiab] OR "Migraine”[tiab] OR "Tension-Type Headache"[Mesh] OR "Tension-Type Headache"[tiab] OR "Central Nervous System Neoplasms"[Mesh] OR "Central Nervous System Neoplasms"[tiab] OR “CNS cancer”[tiab] OR "Brain Neoplasms"[Mesh] OR "Brain Neoplasms"[tiab] OR "Brain cancer"[tiab])
